# Surgical Frontiers: A Comparative Review of Robotics Versus Laparoscopy in Gynecological Interventions

**DOI:** 10.7759/cureus.49752

**Published:** 2023-11-30

**Authors:** Nainita Patel, Kamlesh Chaudhari, Garapati Jyotsna, Jalormy S Joshi

**Affiliations:** 1 Obstetrics and Gynaecology, Jawaharlal Nehru Medical College, Datta Meghe Institute of Higher Education and Research, Wardha, IND

**Keywords:** patient outcomes, emerging technologies, comparative analysis, robotic surgery, laparoscopy, gynecological surgery

## Abstract

This review comprehensively examines the current state and future directions of gynecological surgery, focusing on the comparative analysis of laparoscopy and robotic surgery. The overview highlights the evolution of these surgical techniques, emphasizing their impact on patient outcomes, procedural efficiency, and safety profiles. The analysis encompasses critical factors such as cost-effectiveness, learning curves, and implications for postoperative recovery. The future of gynecological surgery is envisioned through emerging technologies, including augmented reality, single-incision laparoscopy, and artificial intelligence. The coexistence of laparoscopy and robotics is explored, acknowledging their respective strengths and roles in shaping women's healthcare. In conclusion, the dynamic nature of the field is underscored, emphasizing the need for a patient-centered and adaptable approach. Collaboration between healthcare professionals, engineers, and researchers is pivotal in unlocking these innovations' full potential, ensuring continued advancements in gynecological surgery for improved outcomes and enhanced patient care.

## Introduction and background

Advancements in surgical techniques have been pivotal in enhancing the landscape of medical interventions, offering new possibilities and improved patient outcomes. These advancements in gynecology have been particularly transformative, reshaping how practitioners approach various procedures. This section provides a foundational exploration of the evolution of surgical techniques in gynecology, emphasizing the continuous quest for innovation and improved patient care [1].

Over the years, gynecological surgery has witnessed a remarkable evolution, transitioning from traditional open procedures to more minimally invasive approaches. Technological innovations have played a crucial role in this transformation, with laparoscopy and robotics emerging as critical milestones. These advancements aim to enhance the precision and effectiveness of procedures, reduce patient discomfort, and expedite recovery times [2].

The significance of surgical innovations in gynecology extends beyond the operating room. For many patients, gynecological conditions necessitate surgical intervention, and the choice of technique can profoundly impact their overall well-being. Minimally invasive approaches such as laparoscopy and robotic surgery have garnered attention for their potential to minimize scarring, decrease postoperative pain, and shorten hospital stays. Understanding the importance of these innovations is integral to appreciating their implications for healthcare providers and patients [3].

In light of the diverse surgical options available in gynecology, this comparative review seeks to provide a comprehensive analysis of two prominent techniques: laparoscopy and robotics. By juxtaposing these approaches, the aim is to offer insights into their strengths, weaknesses, and applications. Additionally, this review addresses critical aspects such as cost-effectiveness, training requirements, and patient outcomes, guiding practitioners and healthcare decision-makers in making informed choices regarding surgical interventions in gynecology. Through a thorough examination of the available literature and case studies, this review aims to contribute to the ongoing dialogue surrounding the optimal utilization of surgical innovations in gynecological practice.

## Review

Laparoscopy in gynecology

Definition and Principles of Laparoscopy

Apart from emphasizing the avoidance of large incisions, it is crucial to underscore that laparoscopy eliminates the necessity to expose the abdominal/pelvic cavity externally during surgery. Furthermore, the potential for superior aesthetic outcomes associated with laparoscopic procedures is worth highlighting. This is particularly relevant considering the versatility of laparoscopy, which extends beyond complex interventions to include simple diagnostic procedures. A minimally invasive approach in laparoscopy involves inserting a slender, illuminated tube equipped with a camera (laparoscope) through small incisions in the abdomen. This technique gives surgeons a detailed, real-time view of the pelvic anatomy on a monitor. By circumventing the need for extensive incisions, laparoscopy minimizes trauma, expedites recovery, and offers enhanced aesthetic benefits [2].

Common Laparoscopic Procedures in Gynecology

Diagnostic laparoscopy: Diagnostic laparoscopy is a crucial tool in gynecologists' arsenal for assessing and diagnosing a spectrum of pelvic conditions. Through this minimally invasive procedure, surgeons gain direct access to the pelvic cavity, allowing for a comprehensive visual inspection of reproductive organs. The procedure enables the identification of abnormalities and provides a means to determine the underlying causes of symptoms such as pelvic pain or infertility. As an alternative to traditional exploratory surgery, diagnostic laparoscopy minimizes invasiveness, accelerates recovery, and plays a pivotal role in the early and precise diagnosis of various gynecological conditions [4].

Laparoscopic hysterectomy: Laparoscopic hysterectomy represents a revolutionary approach to the removal of the uterus, characterized by small incisions in the abdomen and the utilization of laparoscopic instruments. This technique offers distinct advantages over traditional open hysterectomy, including reduced blood loss, shorter hospital stays, and faster recovery times [5]. Laparoscopic hysterectomy finds application in the treatment of diverse conditions such as uterine fibroids, endometriosis, and certain cancer cases. The procedure minimizes physical trauma, enhances postoperative comfort, and expedites the return to normal activities, thereby improving the overall patient experience [5].

Laparoscopic myomectomy: Laparoscopic myomectomy stands as a refined surgical intervention designed for the removal of uterine fibroids while preserving the uterus. Through small incisions, surgeons achieve precise removal of fibroids, minimizing impact on surrounding tissues. Patients undergoing laparoscopic myomectomy commonly report experiencing less postoperative pain and a faster return to regular activities compared to traditional myomectomy procedures. The minimally invasive nature of this approach contributes to reduced scarring, shorter hospital stays, and improved overall patient satisfaction. Laparoscopic myomectomy exemplifies the progress in gynecological surgery toward procedures that prioritize both efficacy and patient well-being [6].

Advantages and Limitations of Laparoscopy

As a minimally invasive surgical technique, laparoscopy offers several advantages that have transformed the landscape of gynecological procedures. First and foremost, the method significantly reduces scarring, as small incisions result in minimal tissue disruption compared to traditional open surgery. This enhances the cosmetic outcome and mitigates the risk of postoperative complications associated with larger incisions. Moreover, patients undergoing laparoscopic procedures often benefit from faster recovery times, with shorter hospital stays being a hallmark of this approach. The minimally invasive nature of laparoscopy contributes to less postoperative pain, promoting a more comfortable recovery experience. One of the key advantages lies in the improved visualization afforded by the laparoscope, which provides surgeons with a magnified and detailed view of pelvic organs and allows for precise and intricate maneuvers during surgery [7].

However, laparoscopy presents specific challenges that should be acknowledged. Like any surgical skill, laparoscopic techniques require a learning curve, albeit a steeper one compared to laparotomy. This demands specialized training for surgeons to achieve proficiency in handling laparoscopic instruments and navigation. It is important to note that the initial investment in laparoscopic equipment can pose a financial challenge, with costs exceeding those associated with traditional surgical tools. Moreover, the absence of tactile feedback in laparoscopic procedures, relying primarily on visual and mechanical cues rather than direct touch, can be a notable consideration. To clarify, rather than an absolute absence, it would be more accurate to describe this as a reduction or minimization of tactile sensation, which may necessitate adaptation [7,8]. It is imperative to highlight scenarios where laparoscopy may face greater challenges. For instance, intra-abdominal adhesions, particularly in cases with a history of previous abdomen-pelvic surgeries, can significantly complicate laparoscopic procedures, making them technically demanding. In such situations, the need for open surgery may arise to ensure optimal patient outcomes. Considering these factors, healthcare providers must determine the appropriateness of laparoscopy in specific gynecological cases, thereby ensuring comprehensive and individualized patient care [8].

Case Studies Illustrating Successful Laparoscopic Interventions

There are several case studies available that illustrate successful laparoscopic interventions. For example, a single-center analysis of 748 consecutive cases found that laparoscopy is safe and feasible in many cases and is associated with improved outcomes versus laparotomy [9]. Another study reported an overall complication rate of 4.4% for 2,407 laparoscopic procedures, with a re-intervention rate of 0.8% and a mortality rate of 0.08% [10]. Additionally, patient case studies that describe successful laparoscopic hysterectomy procedures are available [11]. While these studies provide evidence of successful laparoscopic interventions, it is essential to note that each case is unique, and outcomes may vary depending on the individual patient and the specific procedure.

Robotics in gynecological surgery

Overview of Robotic Surgery Systems

Robotic surgery systems represent a cutting-edge advancement in gynecological surgery, combining the precision of computer technology with the dexterity of robotic arms controlled by skilled surgeons. These systems typically consist of a console where the surgeon sits, a robotic cart with arms equipped with surgical instruments, and a high-definition three-dimensional (3D) camera providing a detailed view of the surgical site. The surgeon operates the system's controls, translating their movements into the robotic arms' precise actions [12].

Applications of Robotics in Gynecology

Robotic-assisted hysterectomy: Robotic-assisted hysterectomy signifies a significant advancement in gynecological surgery involving the removal of the uterus with the aid of a robotic surgical system. This innovative approach amplifies the precision and maneuverability of surgeons, allowing for the execution of complex tasks with heightened ease. The robotic system's enhanced dexterity proves advantageous in cases demanding intricate dissections, as seen in treating specific gynecological cancers. By leveraging robotic assistance, surgeons can navigate challenging anatomies with precision that might be challenging with conventional surgical approaches, potentially leading to improved surgical outcomes and reduced postoperative complications [13].

Robotic myomectomy: Robotic myomectomy represents a minimally invasive procedure tailored for the removal of uterine fibroids while preserving the uterus. Parallel to the approach employed in robotic-assisted hysterectomy, this method grants surgeons enhanced control and visualization. The robotic arms, guided by a skilled surgeon, perform meticulous tissue dissections, reduce blood loss, and facilitate a swifter patient recovery. The precision afforded by robotic technology in myomectomy minimizes the impact on surrounding tissues. It underscores the potential for improved patient comfort and satisfaction, reinforcing the role of robotics in advancing the field of gynecological surgery [14].

**Figure 1:**
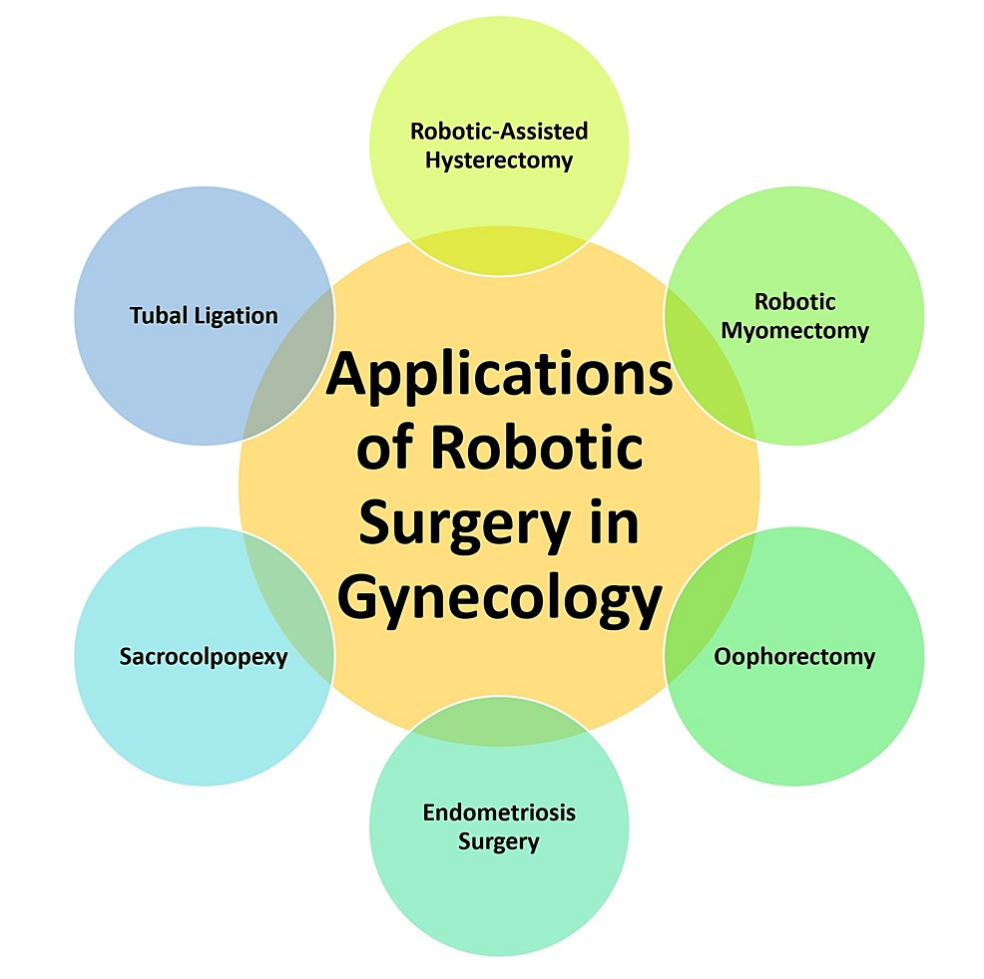
Applications of robotic surgery in gynecology

Other robotic gynecological procedures: The application of robotic surgery in gynecology extends beyond hysterectomy and myomectomy to encompass a spectrum of procedures, showcasing the versatility of this technology. Procedures such as oophorectomy, involving removing one or both ovaries, benefit from the precision and enhanced visualization of robotic assistance. In endometriosis surgery, the robotic system facilitates the precise excision of endometrial tissue, offering a meticulous and targeted approach. Sacrocolpopexy, a procedure addressing pelvic organ prolapse, is refined through robotic technology, providing surgeons with advanced tools for effective repair. Additionally, robotic-assisted tubal ligation offers a modern and minimally invasive means of achieving permanent contraception. The expansion of robotic applications in gynecology reflects a transformative shift toward increasingly specialized and patient-focused interventions, further establishing the role of robotics in advancing the field of women's healthcare [15]. Figure 1 shows the applications of robotic surgery in gynecology.

Advantages and Challenges Associated with Robotic Surgery

Robotic surgery offers several advantages over traditional surgery, including greater precision, enhanced dexterity, and improved visualization [16,17]. Robotic platforms allow surgeons to perform complex procedures through tiny incisions, reducing blood loss, less pain, and faster patient recovery [17]. Additionally, robotic surgery can be used to treat a wide range of conditions affecting the heart, digestive system, bladder, prostate, and more [18]. However, robotic surgery has several challenges, including the technology's high cost, movement latency, and a steep learning curve for surgeons [16]. Potential mistakes can also occur during robotic surgery, and there is a risk of complications such as infection and other surgical site complications [19]. Furthermore, robotic surgery may only be an option for some, and healthcare providers must carefully weigh the pros and cons of robotic surgery to determine whether the benefits outweigh the drawbacks [18].

Case Studies Demonstrating the Efficacy of Robotic Interventions

There are several case studies available that demonstrate the efficacy of robotic interventions in various fields. A pilot randomized controlled trial was conducted to determine the efficacy of a motivational intervention by an autonomous robot, which showed promising results [20]. Another study investigated the effectiveness of robot interventions for cognitive and psychological outcomes among older adults with cognitive impairment and found that robot interventions were effective in improving cognitive and psychological outcomes [21]. Additionally, a case study was conducted to evaluate the effectiveness of a robot-assisted psychological intervention for children with autism spectrum disorder, which showed positive results [22]. While these studies provide evidence of the efficacy of robotic interventions, it is essential to note that each case is unique, and outcomes may vary depending on the individual patient and the specific intervention being performed.

Comparative analysis

Cost-Effectiveness of Laparoscopy vs. Robotic Surgery

Cost considerations play a significant role in the selection of surgical techniques. Laparoscopy is generally regarded as a more cost-effective option compared to robotic surgery. The initial investment for laparoscopic equipment is typically lower, and the disposable instruments used in laparoscopy are often more affordable. In contrast, the high upfront costs of acquiring and maintaining robotic systems can hinder their widespread adoption. However, long-term cost analyses may need to account for reduced hospital stays and faster recovery times associated with robotic surgery [23].

Several studies have shown that robotic-assisted surgery is associated with higher costs than laparoscopy. Contributing factors include the initial purchase of the robot by the healthcare institution, maintenance costs, disposable materials, and longer operative times [24]. In gynecologic oncologic indications, the total extra cost of using the robot was reported to be €1456 per intervention, based on an average number of 165 surgical cases performed per year with the robot [25]. A retrospective analysis of minimally invasive general and bariatric procedures found an average cost of $7,675 for laparoscopic surgery versus $9,436 for robot-assisted surgery, with a contribution margin of $7,761 for laparoscopic versus $3,473 for robot-assisted surgery [26]. A cost-effectiveness evaluation of laparoscopic versus robotic minimally invasive colectomy found that despite comparable or improved clinical outcomes, the overall cost of robotic procedures is higher by as much as $1000 to $2000 per hospitalization [27]. A national analysis of cost disparities in robotic-assisted versus laparoscopic abdominal operations reported an average hospitalization cost of $16,000 for laparoscopic cases versus $18,300 for robotic-assisted cases [28]. A cost-effectiveness evaluation of laparoscopic versus robotic minimally invasive colectomy found that robotic surgery was more expensive than laparoscopic surgery, with an incremental cost-effectiveness ratio of $4,174,849/Quality-Adjusted Life Year (QALY) [27]. These findings suggest that robotic surgery may offer particular advantages, such as improved clinical outcomes, but it is associated with higher costs than laparoscopic surgery. Choosing between laparoscopy and robotic surgery should consider the balance between clinical benefits and cost-effectiveness.

Learning Curve and Training Requirements for Surgeons

The learning curve for mastering laparoscopic techniques is generally shorter than for robotic surgery. Surgeons familiar with traditional open procedures may transition to laparoscopy more seamlessly. In contrast, robotic surgery requires specialized training due to the unique skills to manipulate the robotic console. Training programs are essential to ensure surgeons develop proficiency in operating robotic systems, and ongoing education is crucial to maintaining competency. The learning curve may impact the initial adoption of robotic surgery in some healthcare settings [29]. A recent systematic review of laparoscopic colectomy found that surgeons needed to perform at least 87 procedures to see a meaningful reduction in blood loss and up to 152 to see a meaningful reduction in the conversion rate to an open approach [7].

Patient Outcomes and Postoperative Recovery

Comparing patient outcomes between laparoscopy and robotic surgery involves assessing factors such as postoperative pain, length of hospital stays, and overall recovery. While both techniques generally offer quicker recovery and reduced postoperative pain compared to open surgery, studies suggest that robotic surgery may provide some advantages in specific cases. Patients undergoing robotic procedures, particularly hysterectomy and myomectomy, have reported less pain and shorter hospital stays. However, the overall impact on long-term outcomes and quality of life requires further investigation [30].

Laparoscopic surgery is associated with faster postoperative recovery, less pain, and shorter hospital stays than traditional open surgery [31,32]. Laparoscopic surgery is safe and effective in various procedures, including colorectal surgery [32,33]. Patient-reported outcomes are essential for evaluating the quality of postoperative recovery, and measures such as the Postoperative Quality of Recovery Scale (PQRS) have been developed to assess these outcomes [34]. In some cases, robotic surgery has been associated with improved clinical outcomes compared to other techniques, but it may also be associated with longer operating room times and higher costs [34]. These findings suggest that laparoscopic surgery offers several advantages regarding postoperative recovery and patient outcomes, including faster recovery and less pain. However, robotic surgery may offer improved clinical outcomes in some cases. The decision to choose between laparoscopy and robotic surgery should consider the balance between clinical benefits, cost-effectiveness, procedural efficiency, and the specific context of the surgical intervention.

Procedural Efficiency and Time Considerations

Procedural efficiency and time considerations are crucial in determining the practicality of surgical techniques. Robotic surgery often enhances precision and maneuverability, potentially reducing surgical time. However, robotic systems' setup time can be longer than laparoscopy, which may impact procedural efficiency, especially in cases where time is a critical factor. On the other hand, laparoscopy is known for its shorter setup times and may be more time-efficient for specific straightforward procedures [35].

Robotic surgery has been associated with more extended operating room time than laparoscopy [36]. However, some studies have shown that robotic surgery can be more efficient in specific procedures such as colorectal surgery [36]. Surgeon perception of factors affecting the efficiency of conventional and robotic laparoscopy has been studied, and factors such as instrument availability, instrument malfunction, and patient factors were found to affect efficiency in both approaches [37]. Robotic surgery offers numerous advantages compared to non-robotic minimally invasive surgery, including better field visualization, dexterity benefits, operator ergonomics, and, in some cases, improved clinical outcomes compared to other techniques [38].

Safety and Complication Rates in Laparoscopy and Robotic Surgery

Laparoscopy and robotic surgery are generally associated with lower complication rates and improved safety compared to traditional open surgery. However, the safety profiles of these techniques may vary depending on the procedure and surgeon's expertise. Complications in laparoscopy may include injury to surrounding structures or gas-related complications, while robotic surgery complications may arise from system malfunctions or technical errors. Research is ongoing to comprehensively evaluate each technique's safety and complication rates, considering factors such as the surgeon's experience and patient characteristics [39].

Laparoscopy for benign indications is associated with similar rates of severe complications compared to open surgery, with a 1.4% incidence of severe complications [40,41]. Tsakos et al. conducted a systematic review and meta-analysis comparing robotic-assisted laparoscopic myomectomy (RALM), conventional laparoscopic myomectomy (CLM), and abdominal myomectomy (AM) [14]. Based on 53 eligible studies, the study assessed various surgical outcomes, including blood loss, complications, transfusions, operation duration, conversion to laparotomy, and hospital stay. RALM outperformed AM in all parameters except operation duration. At the same time, RALM and CLM showed similar performance, with RALM demonstrating advantages in reduced intra-operative bleeding for small fibroids and lower conversion rates to laparotomy. The authors concluded that RALM is a safe and effective approach, continuously improving and potentially surpassing CLM in specific patient subgroups [14]. A published study reported an overall complication rate of 6.9% for laparoscopic surgery [42]. Robotic surgery is generally considered safe with low complication rates, but there are unique risks associated with the robotic system, including the potential for human error and mechanical failure [43]. These findings suggest that laparoscopic and robotic surgery have demonstrated safety in various procedures, with comparable or low complication rates. However, it is essential to consider the specific context of each surgical intervention and the potential unique risks associated with robotic systems.

Future directions

Emerging Technologies in Gynecological Surgery

Augmented reality (AR) and virtual reality (VR): Integrating AR and VR technologies represents a transformative leap in gynecological surgery. These immersive technologies offer surgeons unprecedented insight into patient-specific anatomy during preoperative planning and intraoperative navigation. By visualizing three-dimensional representations of internal structures, surgeons can enhance their understanding of the patient's unique anatomy, leading to more precise decision-making during procedures. AR and VR facilitate comprehensive planning and provide real-time guidance, potentially minimizing risks and improving the overall efficacy of gynecological interventions [44].

Single-incision laparoscopy: The evolution of single-incision laparoscopy signifies a significant stride towards minimizing the invasiveness of gynecological procedures. This technique, characterized by a single entry point, aims to reduce scarring and enhance patient recovery. By consolidating multiple incisions into a single access point, surgeons can decrease postoperative pain and improve cosmetic outcomes. Single-incision laparoscopy not only addresses aesthetic concerns but also promises to reduce the risk of complications associated with multiple entry points, contributing to a more patient-friendly approach in gynecological surgeries [45].

Natural orifice transluminal endoscopic surgery (NOTES): NOTES represents a groundbreaking concept in gynecological surgery by exploring the feasibility of conducting procedures through natural body openings, such as the vagina or mouth. This innovative approach eliminates the need for external incisions, thereby significantly reducing patient trauma and potential complications related to incision sites. NOTES holds the potential to revolutionize the field by offering a scarless alternative, mitigating postoperative pain, and potentially expediting recovery. While technical challenges remain, the pursuit of NOTES reflects a paradigm shift towards further minimizing the impact of surgery on patients, emphasizing a holistic and patient-centered approach in gynecological interventions [46].

Potential Improvements in Laparoscopy and Robotic Systems

Miniaturization of instruments: The ongoing pursuit of miniaturizing laparoscopic and robotic instruments marks a critical advancement in gynecological surgery. This innovation is driven by enhancing maneuverability within confined spaces, allowing surgeons to perform even more precise and intricate procedures. Miniaturized instruments enable finer control and access to anatomical structures that may be challenging to reach with more extensive tools. As a result, this development holds the potential to elevate the precision and effectiveness of laparoscopic and robotic surgeries, contributing to improved patient outcomes and reduced trauma [13].

Haptic feedback: Incorporating haptic feedback into robotic surgery systems represents a significant stride towards providing surgeons with a sense of touch during minimally invasive procedures. This technology aims to bridge the gap between surgery's visual and tactile aspects, enabling surgeons to assess tissue characteristics and manipulate delicate structures more effectively. By simulating the sense of touch, haptic feedback enhances the surgeon's ability to navigate complex anatomies, potentially reducing the risk of inadvertent damage and improving surgical precision and safety [47].

Adaptive robotics: The envisioned future of gynecological surgery includes robotic systems that go beyond mere mechanization and incorporate adaptive technologies. These systems respond dynamically to the surgeon's movements, providing real-time assistance and potentially reducing the learning curve associated with robotic surgery. Adaptive robotics could intuitively adjust to the surgeon's preferences and movements, optimizing efficiency and enhancing the overall user experience. This technological evolution not only streamlines the integration of robotics into surgical practice but also promises to make these advanced systems more accessible to a broader range of healthcare professionals, ultimately benefiting patient care on a larger scale [3].

Integration of Artificial Intelligence (AI) in Surgical Procedures

Machine learning for surgical planning: The integration of machine learning in gynecological surgery heralds a new era in preoperative planning. AI algorithms, capable of analyzing vast datasets of patient information, empower surgeons with invaluable insights. By predicting potential challenges and customizing surgical approaches based on individual patient characteristics, machine learning contributes to more personalized and optimized surgical plans. This innovation promises to enhance decision-making, improve procedural outcomes, and streamline the planning phase to ensure the highest level of precision in gynecological procedures [48].

Intraoperative decision support: AI's real-time capabilities offer a transformative paradigm in gynecological surgery by providing surgeons with dynamic intraoperative decision support. These AI systems can suggest optimal instrument placement, identify critical anatomical structures, and alert surgeons to potential complications during surgery. By acting as a virtual assistant, AI contributes to enhanced situational awareness, potentially reducing the risk of errors and ensuring that surgeons can navigate complex procedures with higher confidence and accuracy [49].

Autonomous surgical systems: The prospect of developing semi-autonomous or fully autonomous surgical systems represents an ambitious frontier in gynecological surgery. Envisioned as AI-driven robots capable of performing specific aspects of surgery under the supervision of a surgeon, these systems could revolutionize the field. While preserving critical human oversight, autonomous surgical systems have the potential to execute routine or intricate tasks with unparalleled precision, contributing to standardized and optimized surgical outcomes [50].

Data analytics for outcome prediction: AI's data analytics capabilities extend beyond the operating room to postoperative care. AI can predict patient outcomes by analyzing postoperative data, enabling surgeons and healthcare teams to tailor care plans more effectively. Anticipating potential complications and tailoring postoperative interventions based on AI-driven predictions enhances patient recovery and contributes to a more proactive and personalized approach to healthcare following gynecological surgery [51].

## Conclusions

This comprehensive review underscores the dynamic landscape of gynecological surgery, emphasizing the pivotal roles played by laparoscopy and robotic surgery in advancing patient care. The comparative analysis has shed light on each technique's nuanced strengths and limitations, considering factors ranging from cost-effectiveness and learning curves to patient outcomes and safety profiles. The future of gynecological surgery holds tremendous promise with the integration of emerging technologies such as AR, single-incision laparoscopy, and AI. These innovations, alongside potential improvements in robotic and laparoscopic systems, are poised to redefine surgical precision, procedural efficiency, and postoperative recovery. The coexistence of these technologies underscores the importance of a nuanced and patient-centered approach, where the choice between laparoscopy and robotic surgery is tailored to individual patient needs, procedural requirements, and the evolving landscape of healthcare resources. As we navigate this era of innovation, a collaboration between medical professionals, engineers, and researchers remains critical to harnessing the full potential of these advancements, ultimately shaping a future where gynecological surgery is characterized by improved outcomes, reduced invasiveness, and enhanced patient well-being.
